# Catalogue of Inorganic Substances Employed in Medicine, with the Powers of Each Substance

**Published:** 1851-01

**Authors:** 


LOUISVILLE, December 1850.

*To the Editors of the Western Medical Journal:—*

The accompanying tabular view of remedial agents was prepared by one of his pupils some years since from the Mss. of Dr. Wm. Tully of New Haven, Connecticut, and formerly Professor of Materia Medica in the Medical Institute of Yale College. Dr. Tully has been justly regarded by all who have known him as one of the most erudite and philosophical scholars in the Medical profession. Unfortunately for science he has not yet published much, (except in the department of Botany) but by his power as a teacher he has uniformly excited the zeal and profound gratitude of all who have ever enjoyed his instructions. With a commendable spirit of liberality his very voluminous manuscripts, covering a wide range of medical and scientific learning, have always been open to his pupils. The present classification of medical agents it is believed is entirely his own, and it will, I am sure, be examined with great interest by those who make such subjects a study. Yours truly, B. S. Jr.

Art. II.—*Catalogue of inorganic substances employed in medicine, with the powers of each substance.* Prepared from the manuscripts of Dr. Wm. Tully, with a system of powers in the order and kind of their operation.

Remedial agents may be classed in three Orders according to the nature of their operation upon the human constitution. Each of the Orders will embrace as many Classes, and each Class three or more groups of powers.

Order I.—Class I.—[1]*Antiphlogistics*, "diminish vital energy and strength of action in the circulating system"—(Tully.) *Antiph.* operate suddenly, but their *effect* is lasting. [2]*Alteratives or "Adenagics" (Tully)* occasion gradual and permanent changes of action and condition in the glandular, or secernent and absorbent systems. [3]*Sedatives* or "*Neuragics*" *(Tully)* occasion slow or more rapid and permanent changes of action and condition in the nervous, and secondarily in the nutritive and circulating systems. [4]*Tonics* increase permanently, or for considerable periods of time, the active functions of organic life, circulation, digestion, &c. They restore, restrain, and augment *tone.*

Class II.—[1]*Diffusible stimulants*, "*Antisbestics*" *(Tully.)* Augment rapidly, but transiently, the activity of circulation and counteract *Antiphlogosis.* [2]*Narcotics* exalt, sustain, and finally *modify and suspend* the action of the nervous system of animal life. They operate secondarily upon the *brain* and upon the nerves of digestion, circulation, &c. [3]*Exhilarants* "*Euphrenics*" *(Tully)* exalt the cerebral functions merely.

CLASS III.— [1]*Cathartics* increase downward peristaltic motion, and augment intestinal mucous secretions; (but the last is not their proper action.) [2]*Emetics* occasion an upward peristaltic movement generally sudden and transient. [3]*Sternutatories* and such agents as excite sudden and powerful action of the respiratory organs; (such are suffocating and irritating gases.)

ORDER II.—CLASS I.— [1]*Styptics* occasion a vital contraction of the organic solids, and suspend secretion from mucous surfaces. [2]*Levatics* (or demulcents) allay irritation of mucous surfaces. [3]*Dieuretics*, *Emmenagogues*, &c. increase particular secretions of mucous surfaces, without irritation.

CLASS II.— [1]*Rubefacients* occasion a temporary relaxation and consequent engorgement of the superficial capillaries, (Errhines and irritants used simply to promote secretion are of the same *power.)* [2]*Epispastics proper*, *irritate*, change organically and destroy the surface. [3]*Mechanical irritants* exalt, irritate, and finally *exhaust* the vitality of the skin and mucous membranes.

CLASS III.— [1]*Fomentations*, *Cataplasms*, *warm baths*, &c. assist, exalt and finally exhaust the organic activity of parts, or of the whole system. [2]*Cold bath*, profuse blood-letting and *shocks* of all kinds, intended to effect sudden and violent revulsions in the organic system. [3]*Regimen.*

ORDER.—REMEDIA INTELLECTUALIA.

[NOTE.—Dr. Tully classes Rubefacients, Epispastics and Mechanical irritants under one term, ORAESTHETIC. *Expectorants* as a class or Power have no existence; many medicines are used to increase expectoration *indirectly*. Sialagogues are either *rubefacient* or Alterative. "Adenagic" (Tully.)

### MATERIA MED. CHEMICA.

1 Acidum, aceticum........ Antiph. Neur. Rubefacient.
2 " arsenicum ........Ton. Aden. Neur. (or Sed.)
3 " arsenosum........ " " "
4 " auricum........ Neur. (or Sed.) Adeu. (or Alterative.
5 " boracicum........ " Antiph. Oræsth. (or Irrit.)
6 " carbonicum...... " " "
7 " chlorauricum.... Adenag.
8 " chloroferricum...... " Tonic.
9 " chlorohydrargyricum..Neur. " Emet. Rubefacient.
10 " chlorohydrargyrosum...." " Cathartic, (Calomel.)
11 " chlorohydricum.........." " Antiph. Oræsthetic.
12 " chloroxynitricum....... " " " "
13 " citricum................" " "
14 " cyanauricum.......... " " Narcot. "
15 " cyanohydrargyricum...." " " Emetic.
16 " cyanohydricum.......Pure Narcotic.
17 " fluoboricum..........Escarotic. (*Oræsthetic.*)
18 " fluohydricum............. "
19 " hypantimoniosum....Antiph. Emet Cath. Neur. Irrit.
20 " hypocarbonicum........" " "
21 " iodohydrargyricum..Aden. Neur. Cath. (Calomel.)
22 " iodoplumbicum..... " " Suboraesthet.
23 " lacticum...........Antacid.
24 " malicum............Antiph. Neur. Oræsth.
25 " nitricum............ " " " Aden.
26 " oxalicum........... " " " "
27 " phosphoricum...... " " " "
28 " succinicum.........Narcotic Euphrenic.
29 " sulpharsenicum....Neur. Aden. Oræs. Subcath. Emetic.
30 " sulpharsenosum...(Realgar) " " Neur. Emet. Subcath.
31 " sulphuricum......Antiph. " Oræsthetic.
32 " tartaricum........ " " "
33 Æther aceticus..........Exhilarant, Irritant, Subantiphlog.
34 " bromohydricus.... " " "
35 " chlorohydricus.... " " "
36 " citricus...... .... " " "
37 " hydricus.......... " " "
38 " hypocarbonicus (Seur Oxalicus) Exhil. " "
39 " hyponitrosus " " "
40 " iodohydricus " " "
41 " malicus " " "
42 " sulphuricus " " "
43 " tartaricus " " "
44 Alcohol, ...............Narcotic, Euphren. Antisbestic, Diaphoretic.
45 " dilutum...........Exhilarant.
46 " purum.......... "

47 Aluminæ et Potassæ, Sulphas. Styp. Antiph. Neur. Suboræs, Subcath. [Subemetic.
48 Ammonia alkalina............Subantiph. Oræsth. Emetic.
49 Ammoniæ Acetas............Antiph. Neur. Laxat.
50 " alkalina aqua............ (see Carbonas.)
51 " " spiritus............ "
52 " auras..............Adenag. Neurag.
53 " bicarbonas..........Oræsth. Subantiph. Emet.
54 " bichlorohydrargyras..Neur. Aden. Cath.
55 " carbonas............Oræsth. Subantiph. Emet.
56 " citras..............Antiph. Neur. Laxat.
57 " dichlorohydrargyras, (White Precipit. of M.) Aden. Neur. Emet. [Epispastic.
58 " disarsenas..........*Tonic.* Aden. Neur. Subemet. Subcath.
59 " disarsenis.......... " " " " "
60 " hemiolochloroferras. " " " " "
61 " hypercarbonas.......Antiph. Neur. Laxat.
62 " malas............. " " "
63 " oxalas............ " " "
64 " sesquicarbonas.....Oræsth. Subantiph. Emet.
65 Ammonii chloridi bichlorohydrargyras ..Aden. Episp. Emet. Neur.
66 " sulphidi sulphohydris...........Neur. Aden.
67 " sulphidum.................. " "
68 Antimonii deutochloridum..Anti Emet. " Laxative et Oræs.
69 " perchloridum.....Antiph. " Emet. Oraesthetic.
70 " et potassae tartras.. " Emet. " Oræsth. Laxat.
71 " sesquichloridum.... " " Emet. Oræs.
72 " sesquioxidum....... " Emet. Coxat. Neur. Oræsth.
73 Aqua fervens............Epispastic.
74 " et spiritus ammon. alkal... (see Ammoniæ.)
75 Argenti nitras..............Oræsthetic. *(Not Tonic.)*
76 Arsenici protosulphidum....(Orpiment,) Aden. Neur. Oræsth. Subcath. [Emet.
77 " sesquisulphidum, (Realgar.) Neur. Aden. Subemet. Subcath.
78 Auri cyanas..................... Aden. Neur. " "
79 " fulminas.................. " " " "
80 " protocyanidum............ " Narcot. Neuragic.
81 " sesquichloridum........... " Neur. Subemetic. Subcath.
82 " sesquioxydum............. " "
83 " trisiodidum............... " " Suboræsthetic.
84 Aurum elementarium............. Mech. Irritant.

B

85 Baryti chloridum...........Aden. Neur.
86 Baryti iodidum........... " " Suboræsthetic.
87 Beurilli hydroquretum.....Pure Narcotic. Essential oil of bitter almonds.
88 Bismuthi tetrakinitras.....Pure Neuragic. (Pearl White.)
89 Brominum elementarium..Neur. Aden.

### C

90 Calciæ acetas.......Antiph. Neur. Laxat.

91 " binacetas.... " " "

92 " carbonas....Antiphlogistic. Antacid.

93 " et Magnesiæ carbonas.

94 " Phosphato-Antimonis ....Anti. Emet. Laxative. Neur. Oræst.

95 Calcii chloridum................Aden. Neur. Subantiph. Oræs.

96 " chloroxydum............ " " "

97 " deutiodidum............ Neur. Aden. "

98 " protiodidum............ " " "

99 " protoxydum.............Oræs. Subantiphlog.

100 Carbo lignarius...............Absorbent, Mechanical irritant.

101 Carbonis sesquichloridum..........Oræsth. Subantiph. Euph.

102 " dinitroquretum...........Narcotic.

103 " deutosulphidum.........Oræsthetic. Subantiph. Euphren.

104 Chlorinum elementarium.........Aden. Neuragic.

105 Creasotina.....................Pure Oræsthetic or Irritant.

106 Cupri et Ammoniæ Sulphas..Neur. Styptic. Emet. Cath. Oræsthetic.

107 " diprotacetas........... " " " " "

108 " disiodidum............. " Aden. " Epispastic.

109 " hemioloprotacetas..... " Styptic. " Subcath. Oræsth.

110 " protacetas............. " " " " "

111 " protiodidum...........Oræs. Neur. " " Aden.

112 " protosulphas...........Neur. Styptic. " Cath. Oræsth.

113 " subprotacetas.......... " " " " "

114 " triprotacetas........... " " " " "

115 Cuprum ammoniatum.

116 Cyanogenii protiodidum.........Narcot. Aden. Neur.

117 Cyanogenium..................Narcotic.

### F

118 Ferri protiodidum.........Aden. Neur. Subemet. Subcath. *Tonic.*

119 " protocarbonas...... Tonic.

120 " protochloridum.....Adenagic. *Tonic.*

121 " protophosphas......Tonic.

122 " protosulphas........ "

123 " protoxydum......... "

124 " sesquichloridum....Aden. *Tonic.*

125 " sesquiodidum...... " " Neur.

126 " sesquioxydum......Tonic

127 Ferrum elementarium......Mechanical Irritant.

### H

128 Hydrargyri dichloridum......(Calomel.) Neur. Aden. Cath.

129 " diprotosulphas......(Aden.) Used as an Errhine.

130 " disiodidum..........Cath. Neur. Aden.

131 " disoxydi dinitras.... " " "

132 " disoxydum..........Neur. Cath. Adenag.

133 Hydrargyri oxydum rubrum....("Chemical Irritant.") Aden.
134 " præcipitatum album.. " " Oræsth.
135 " protacetas............Aden. Neur. Emet. Epispastic.
136 " protiodidum.........." " Cath.
137 " protochloridum.......(Corros. Sublim.) Aden. Emet. Epispastic. [Neur.
138 " protocyanidum.......Neur. Aden. Narcot. Emet.
139 " protoxydum.......(Red Precipitate.) Aden. Neur. Emet. Epispas.
140 " subiodidum.......Cath. Neur. Aden.
141 Hydrargyrum elementarium..Mech. Irritant.
142 " sublimatum carrosivum. (see 137.)
143 Hydrogenii dentoxydum.....Escarotic.
144 " dicarbureti dentochloridum (Chloric Æther).. Irrit. Subantiph. [Euphraenim.

I

145 Iodinum elementarium.....Aden. Oræsth. Neur.

M

146 Magnesiæ carbonas.......Antiph. (Antacid.)
147 " citras............Antiphlog. Neur. Laxat.
148 " hypercarbonas.. " (Antacid.)
149 " malas.......... " Neur. Laxat.
150 " oxalas.......... " " "
151 " sulphas.......... " " Cath.
152 Magnesii oxydum....... " (Antacid.)
153 Morphinæ arsenas........Narcot. Aden. Neur. (Tonic?)
154 " sulphas......... " Antisbestic. Euphrenic. Diaphoretic.

N

155 Nitrogenii protoxydum.......Pure Euphrenic or Exhilarant.

O

156 Oleum animale empyreumaticum.....Pure Exhilarant.
157 " succini electri.............. " "

P

158 Phosphorus elementarius.......Antisbæstic. Oræsthetic.
159 Plumbi diprotacetas............Styptic. Neurag.
160 " protacetas............. " "
161 " protiodidum............Neur. Adenagic.
162 " protocarbonas......... "
163 " protocyanidum........ " Narcotic.
164 " protoxydum.......... " Adenagic.
165 " triprotacetas...........Styptic. Neuragic.
166 Potassæ acetas................Anti Neur. Laxat.
167 " bicarbonas...........Antiph. " Oræsth.
468 " bihypercarbonas....... " " Laxat.
169 " binoxalas............. " " "
170 " bitartras............. " Cath. Neur.
171 " et calciæ silicas.......Mechanical Irritant.

172 Potassæ carbonas.............Anti. Oræsth. Neurag.
173 " chloras.............Antiph. " "
174 " chloris............. " " "
175 " citras............. " Neur. Laxat.
176 " disarsenas...........Neur. Alter. Tonic.
177 " disarsenis.......... " " "
178 " hypercarbonas.....Ant. Neur. Laxat.
179 " malas.............Anti. " "
180 " nitras............ " Oræsth. Antiph.
181 " oxalas.............Anti. " " Laxat.
182 " sesquicarbonas.... " Anti. et ?.
183 " et sodæ tartras....Anti. Cath. Neur.
184 " sulphas.......... " " "
185 " tartras .......... " " "
186 Potassii chloroxydum.....Aden. Subantiph. " Oræsth.
187 " cyanidi cyanauras..Narcot. Aden. "
188 " (cyanidi) cyanohydrargyras.. " Narcot. Emet. Neur
189 " deutiodidum................Neur. Subantiph.
190 " iodidi iodohydrargyras......Aden. Cath. Neur. Emmenagogic.
191 " protiodidi iodoplumbas...... " "
192 " iodidum ..................Neur. Aden. Subantiphlog.
193 " protocyanidum.............Oræsth. Narcot. "
194 " protoxydum............. " "

Q

195 Quinini cyanidi dicyanoferris....Tonic. Narcotic.
196 Quininæ sulphas.................*Pure* Tonic. (?.)

S

197 Sodæ acetas.................Anti. Neur. Laxat.
198 " biboras...............Antiph. Neur. Oræsth.
199 " bicarbonas............Antiph. Neurag. Oræsth.
200 " et calciæ silicas ........Mechanical irritant.
201 " carbonas.............Anti. Neur. Oræsth. (Barilla.)
202 " citras.................Neur. Laxat. Antiphlogistic.
203 " diphosphas............ Neur. Antiph. Catch.
204 " hypercarbonas.........Neur. Laxat. Anti.
205 " malas.................Neur. Lax. Antiph.
206 " nitras................Anti. Neur. Laxative.
207 " oxalas............... " " "
208 " sesquicarbonas.........Antiph. Neur. Oræsth.
209 " sulphas.................Neur. Antiph. Cath.
210 Sodii deutiodidum ... ......Neur. Adenagic. Subantiph.
211 " chloridi chlorauras......Neur. Aden. Narcot. Subemetic. [Subcathartic.
212 " chloroxydum............Oræsth. Aden. Neuragic. Subantiph.
213 " protiodidum.... ....Neur. Aden. Subantiphlog.
214 " protoxydum............Oræsth. (Caustic.) Subantiphlogistic.

215 Spiritus vini Gallici..........Antisbestic. Narcotic.
216 Stanni deutochloridum.......Pure irritant.
217 " protochloridum........ " " (Oræsthetic.)
218. Stannum elementarium.....Mechanical irritant.
219 Strychniae sesquiarsenas.....Aden. Neur. Narcotic.
220 Sulphur elementarium.......Neur. Aden. Cathartic.
221 " sublimatum.......... " " "
222 Sulphuris protiodidum.......Neur. Suboræsthetic. Adenagic.

V

223 Vinum vitis viniferæ........Antisbestic. Narcotic.

Z

224 Zinci pentakiprotacetas.......Styp. Emet. Catch. Neur.
225 " protacetas............Styp. Emet. Cath. Neur.
226 " protiodidum...........Neuragic. Alterative.
227 " protocarbonas..........Neur. pura et Alter. (Lapis Calimin.)
228 " protochloridum.......Pure Oræsthetic or Irritant.
229 " protocyanidum.........Neur. et Narcoticum.
230 " protosulphas...........Styp. Emet. Cath. Neur.
231 " protoxydum............Neur. et Alterat.
232 " sulphas................Epispastic. Styptic. Emet. Cath.

---

### Table of Powers and their Symbols.

( 1. ) Antiphlogistic..........................Symbol...Antiph.
( 2. ) "Adenagic" or *Alterative*..................."....Aden. or Alter.
( 3. ) Sedative or "Neuragic"..................."....Sed. or Neur.
( 4. ) Tonic ......................................"....Ton.
( 5. ) Diffusible Stimulants. "Antisbestics" ......."....Antisb. or Diff. Stim.
( 6. ) Narcotics ................................."....Narc.
( 7. ) Exhilarants or "Euphrenics".............."....Euph.
( 8. ) Cathartics, Laxatives, &c. ................"....Lax. Cath.
( 9. ) Emetics ...................................."....Emet.
(10. ) Agents which stimulate the respiratory system.
(11. ) Styptics................................Symbol..Styp.
(12. ) Levatics or Demulcents. (Vegetable) ......"....Lev.
(13. ) Diuretics, Emmenagogues, &c. " ........."....Diuret. Emmen.
(14. ) Rubefacients, "Oræsthetic '....".........."....Rub. or *Orœs*.
(15. ) Epispastic. Escarotic..........".........."....Epis. Escar. or *Orœs*.
(16. ) Mechanical Irritant............".........."....Mech. Irrit.

